# Proximal ROw carpectOmy versus four-corner Fusion (PROOF-trial) for osteoarthritis of the wrist: study protocol for multi-institutional double-blinded randomized controlled trial

**DOI:** 10.1186/s13063-023-07544-1

**Published:** 2023-08-07

**Authors:** Mikko Alanen, Susanna Stjernberg-Salmela, Eero Waris, Teemu Karjalainen, Jouko Miettunen, Jorma Ryhänen, Samuli Aspinen

**Affiliations:** 1 Department of Hand Surgery, Helsinki University Hospital, Helsinki, Finland; 2 Department of Hand Surgery, Central Hospital of Central Finland, Jyväskylä, Finland; 3 Center for Life Course Health Research, University of Oulu, Oulu, Finland

**Keywords:** Arthrodesis, Arthroplasty, Osteoarthritis, Randomized controlled trial, Wrist injuries

## Abstract

**Background:**

Scapholunate advanced collapse (SLAC) and scaphoid non-union advanced collapse (SNAC) are common types of wrist osteoarthritis (OA). Non-operative treatment consists of pain medication, splinting, and avoiding activities that induce pain. However, in case a course of conservative treatment is unsuccessful, operative treatment is needed. The two most conventional operative approaches for SLAC/SNAC OA are four-corner arthrodesis (FCA) and proximal row carpectomy (PRC). Although FCA is the gold-standard operative technique and may lead to superior grip strength, the evident benefit of PRC is that it obviates any need for hardware removal and controlling for bony union. To date, no high-quality randomized controlled trial comparing FCA and PRC exists. As clinical outcomes seem comparable, a trial that assesses patient-reported outcomes, adverse events, and secondary operations may guide clinical decision making between these two procedures. Thus, the aim of this multi-institutional double-blind randomized controlled trial is to study whether PRC is non-inferior to FCA in treating SLAC/SNAC OA. We hypothesize that PRC is non-inferior to FCA with lower economic expanses.

**Methods:**

The trial is designed as a randomized, controlled, patient- and outcome-assessor blinded multicenter, two-armed 1:1 non-inferiority trial. Patients with SLAC/SNAC-induced wrist pain meeting trial inclusion criteria will undergo wrist arthroscopy to further assess eligibility. Each patient eligible for the trial will be randomly assigned to undergo either FCA or PRC. The primary endpoint of this study is the Patient Rated Wrist Evaluation (PRWE) at 1-year after FCA versus PRC. Secondary outcomes include Quick-Disabilities of the Arm, Shoulder and Hand, EQ-5D-5L, pain, grip strength, wrist active range of motion, radiographic evaluation, and adverse events. Trial design, methods, and statistical analysis plan will be presented here.

**Discussion:**

We present an RCT design comparing FCA vs PRC for SLAC/SNAC-induced OA. The results of this trial will assist in decision making when planning surgery for SLAC/SNAC.

**Trial registration:**

ClinicalTrials.gov NCT04260165. Registered February 7, 2020.

**Supplementary Information:**

The online version contains supplementary material available at 10.1186/s13063-023-07544-1.

## Administrative information

Note: The numbers in curly brackets in this protocol refer to SPIRIT checklist item numbers. The order of the items has been modified to group similar items (see http://www.equator-network.org/reporting-guidelines/spirit-2013-statement-defining-standard-protocol-items-for-clinical-trials/).

**Table:** 

Title {1}	Proximal ROw carpectOmy versus four-corner Fusion (PROOF-trial) for the treatment of osteoarthritis of the wrist: Study protocol for multi-institutional double-blinded randomized controlled trial
Trial registration {2a and 2b}.	ClinicalTrials.gov NCT04260165. Registered February 7, 2020.
Protocol version {3}	Version 5.0, updated: December 17, 2020
Funding {4}	The trial is funded by the State funding for university-level health research (Helsinki University Hospital), Helsinki University Hospital institutional funding for Musculoskeletal and Plastic Surgery, and grants received from The Finnish Society for Surgery of the Hand, the Vappu Uuspää foundation, and the Finnish Medical Foundation.
Author details {5a}	Mikko Alanen^1^, Susanna Stjernberg-Salmela^1^, Eero Waris^1^, Teemu Karjalainen^2^, Jouko Miettunen^3^, Jorma Ryhänen^1^ and Samuli Aspinen^1^ ^1^Department of musculoskeletal and plastic surgery, Helsinki University Hospital, Helsinki, Finland ^2^Department of Hand Surgery, Central Hospital of Central Finland, Jyväskylä, Finland ^3^Department of Biostatistics, University of Oulu, Oulu, Finland
Name and contact information for the trial sponsor {5b}	Department of Musculoskeletal and Plastic Surgery, Helsinki University Hospital, Topeliuksenkatu 5, 00260 Helsinki Tel: + 35,894,711
Role of sponsor {5c}	The sponsor will oversee the conduct of the trial, while ensuring compliance with regulatory bodies and ethical regulations.

## Introduction

### Background and rationale {6a}

Osteoarthritis (OA) of the wrist is a common disorder and can lead to pain and substantial functional impairment. Most cases are due to traumatic sequelae, although atraumatic OA of the wrist may follow similar degenerative patterns. Scapholunate advanced collapse (SLAC) and scaphoid non-union advanced collapse (SNAC) are the most common examples of wrist OA seen in the clinical setting.

Much like long-standing non-union of the scaphoid, attenuation of the scapholunate ligament commonly leads to a secondary development of wrist OA. Both conditions lead to abnormal joint kinematics with a further development of dorsal intercalated segment instability (DISI) deformity and a rotatory subluxation of the scaphoid. These changes initiate degenerative arthritis at the radioscaphoid articulation, followed by carpal collapse and midcarpal arthritis [1]. A four-stage classification of the progressive pattern of wrist OA was first described by Watson et al. [2] The pattern is presented in the Supplement 1 [2–4].

Among active patients, the progression of SLAC and SNAC usually leads to substantial pain and restriction in the range of motion (ROM). Conservative treatment consists of pain medication, splinting, and avoiding painful activities. However, if symptoms worsen with disease progression, operative treatment is preferred [5].

Various operative approaches exist depending on OA stage, patient requirement level, and expert opinion. One of the most common approaches to either SLAC or SNAC I-III OA is four-corner fusion (FCA). This procedure includes scaphoid excision and fusion of the remaining proximal and distal carpal rows (capito-lunate-hamato-triquetral fusion). Another commonly used motion-preserving reconstruction to SLAC or SNAC I-II is proximal row carpectomy (PRC), which includes the excision of the scaphoid, lunate, and triquetrum, creating a neoarticulation between the capitate and lunate fossa of the radius.

Although several studies report the outcomes of PRC and FCA, there is a paucity of high-quality evidence that would support the use of one technique over another. Two RCTs exist comparing these techniques [6, 7]. However, these trials suffer from methodological shortcomings, such as lack of blinding, high risk of selection bias, and incomplete outcome reporting. Generally, studies show no significant difference in ROM, grip strength, pain, or patient-rated outcomes [6–8]. However, some study groups suggest that FCA may be superior in terms of grip strength, while PRC may be preferable in terms of ROM, postoperative complications, and secondary operations [9–12]. Some studies indicate that PRC is more cost-effective [13, 14], while Daar et al. [15] concluded that FCA with cannulated compression screws is the most cost-effective approach. A long-term study reported fewer arthritic changes after FCA versus PRC. However, there was no apparent correlation between radiographic OA and patient satisfaction [16].

The most recent meta-analysis comparing PRC and FCA from Kamir et al. (2020) used flexion/extension ROM, grip strength, and pain level as outcome measures. They found that PRC was statistically but not clinically significantly better than FCA in all outcome measures [17].

Wrist OA due SLAC- and SNAC-degenerative pattern remains a common disorder encountered by a wrist surgeon. To date, most surgeons will choose to manage patients with SLAC/SNAC OA with FCA over PRC. However, the use of PRC has gained popularity among clinicians due its simplicity and predictable results, without the need to remove hardware or control for bony union. We hypothesize that PRC is non-inferior to FCA with lower economic expanses. While superiority of either procedure may be insurmountable to present, a thorough assessment of important considerations, such as AEs, postoperative pain, ROM, and convalescence time, should be examined by means of a randomized controlled trial (RCT). Thus, we planned a study protocol for a multicenter, double-blinded, randomized controlled non-inferiority trial comparing PRC vs FCA for the treatment of SLAC/SNAC I-II OA of the wrist.

### Objectives {7}

This is a multicenter study comparing the outcomes of PRC and FCA for the treatment of SLAC/SNAC I-II wrist OA. We also include patients with non-reconstructible scaphoid non-union or static malalignment of carpal bones after SL disruption. The primary endpoint of this study is the Patient Rated Wrist Evaluation (PRWE) at 1-year after FCA versus PRC. The objectives are listed in Table 1. The study includes a cost-effectiveness analysis.

**Table 1:** Study objectives

**Primary endpoint**
Patient-Rated Wrist Evaluation
**Secondary endpoints**
Quick-Disabilities of Arm, Shoulder, and Hand
Pain (visual analog scale)
Global improvement
EQ-5D-5L
Wrist active range of motion
Grip strength
Adverse events and major adverse events
Progress of arthritic changes
Time to union (FCA group)

### Trial design {8}

**Fig. 1:**
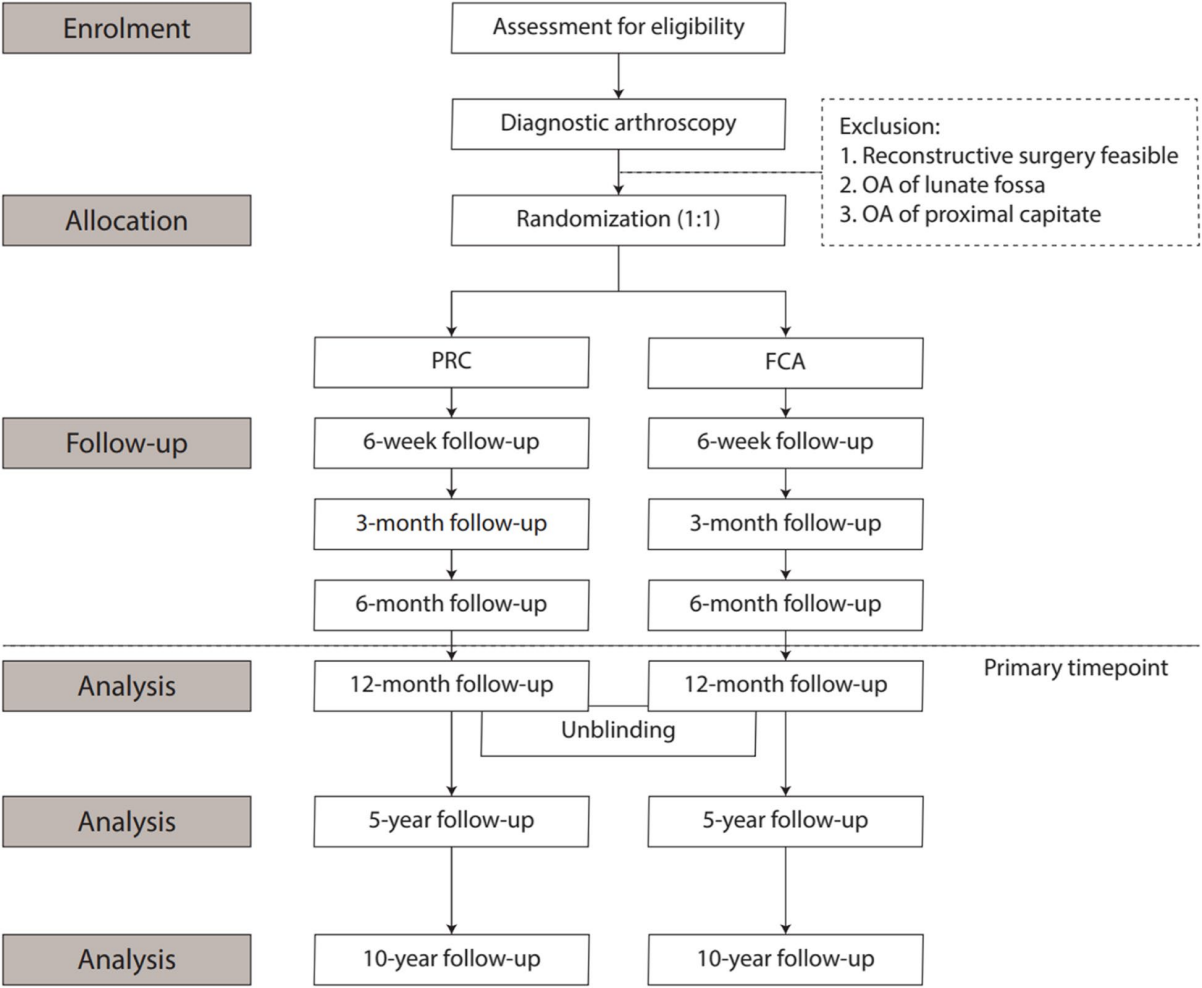
CONSORT diagram of the trial

PROOF is a multicenter, prospective, patient- and outcome assessor-blinded, randomized, controlled two-armed 1:1 non-inferiority trial. The CONSORT diagram of the trial cohort is presented in Fig. 1.

## Methods: participants, interventions, and outcomes

### Study setting {9}

The trial will be conducted in four Finnish hospitals: the Helsinki, Tampere, and Turku university hospitals and Central Finland central hospital.

### Eligibility criteria {10}

We will assess the eligibility of all patients with SLAC/SNAC pattern wrist OA, chronic SL dissociation, and scaphoid non-union referred to the study hospitals. A 3-month course of conservative treatment will be initiated and patients not responding to non-operative treatment will be referred to a recruitment investigator (RI) and screened according to the inclusion and exclusion criteria. RI will confirm the clinical and radiological diagnosis and ensure that an adequate period of conservative treatment is completed without significant response. Patients with a prior attempt to reconstruct scaphoid non-union or SL ligament will be eligible for this trial. If the patient is eligible and agrees to participate, signed informed consent will be provided and baseline data will be collected (Table 2).

**Table 2:** Trial eligibility criteria

Inclusion criteria	Exclusion criteria
1. Patient with: a) Irreducible carpal collapse due to chronic SL ligament injury, with normal joint cartilage, or b) Non-re-constructible scaphoid non-union with normal joint cartilage, or c) SLAC/SNAC I-II OA	1. Patient suitable for: a) SL-ligament reconstruction, or b) Scaphoid reconstruction
2. Pain persisting after 3 months of conservative treatment	2. Ulnocarpal, pancarpal, or advanced lunocapitate OA
3. Age 18–75 years	3. Age < 18 or > 75 years
4. ASA I-II	4. Inflammatory joint disease
5. Sufficient skills in spoken and written Finnish or Swedish	5. Heavy smoking (over 20 cigarettes/day)
	6. Disease or medication attributable to the fusion rate
	7. Alcohol or drug abuse
	8. Unstable psychiatric condition
	9. Symptoms are attributable to another wrist or upper limb condition
	10. Neurological condition affecting upper limb function
	11. Previous operation on the affected upper limb within 6 months

*ASA*, American Society of Anesthesiologists classification

The participant will then be scheduled for a diagnostic wrist arthroscopy prior to randomization and any study procedures to further confirm eligibility. The surgeon will confirm the radiological diagnosis, that the cartilage of the lunate fossa, proximal lunate, and capitate is preserved (Outerbridge 0–2, Table 3) [18] and that both procedures, FCA or PRC, would be indicated in the clinical scenario. If the surgeon deems that SL ligament or scaphoid reconstruction can be performed, the participant is excluded.

**Table 3:** Modified Outerbridge classification. Evaluation of articular cartilage during arthroscopy [18]

Classification	Description
0	Normal articular cartilage
1	Softening of the articular cartilage
2	Fibrillation or superficial fissures of the cartilage
3	Deep fissuring of the cartilage without exposed bone
4	Exposed bone

### Who will take informed consent? {26a}

Patients with SLAC/SNAC OA referred to the study hospitals will be screened for eligibility by RIs. All RIs are consultant level hand surgeons. Patients eligible for this trial will receive written and verbal information. The RI will obtain consent from the participants and collect baseline data prior to randomization. Each patient will be informed that participation in the trial is voluntary and withdrawal is allowed at any time.

### Additional consent provisions for collection and use of participant data and biological specimens {26b}

This trial does not involve collecting biological specimens for storage.

## Interventions

### Explanation for the choice of comparators {6b}

FCA has been the gold standard for treatment of SLAC/SNAC II-III wrist OA. However, the use of PRC has gained popularity among clinicians in SLAC/SNAC I-II OA due its simplicity and predictable results. The evident benefit of PRC is that it obviates the need for hardware removal and control for bony union. However, PRC cannot be performed in stage III OA as the capitolunar surface is involved [19]. In this study, FCA will act as standard treatment and PRC as active control in stage I-II SLAC/SNAC wrist OA.

### Intervention description {11a}

A uniform anesthesia protocol will be used. Brachial plexus blockade is the method of choice with or without general anesthesia to provide good immediate postoperative pain management. Preoperative antibiotic prophylaxis will be used (cefuroxime 1.5 g or clindamycin 600 mg). Each physician performing surgery is a consultant level hand surgeon familiar with both operative techniques used in this study.

The patient will be placed in supine position. A pneumatic tourniquet will be applied. For arthroscopy, the wrist is placed in a traction tower with approximately 10 pounds used for traction to allow visualization. Standard 3–4, 6R, radial and ulnar midcarpal portals will be used. A systematic examination of articular cartilage and extrinsic and intrinsic ligaments will be performed to confirm patient eligibility.

A standard dorsal approach through the third extensor (extensor pollicis longus-, EPL-) compartment will be used for both procedures. The posterior interosseus nerve (PIN) lying in the fourth compartment will be denervated. A capsulotomy will be performed as described by Berger et al. [20]. After capsulotomy, PRC or FCA will be performed according to the randomization. Absorbable sutures will be used for the closure of joint capsule and extensor retinaculum. The EPL tendon will be left outside the extensor retinaculum. Skin will be closed with non-absorbable interrupted sutures. A soft dressing and a short arm splint will be applied.

### Proximal row carpectomy

The detailed surgical technique used for PRC is described by Stern et al. [21]. When removing the proximal carpal bones, care will be taken not to damage the lunate fossa and the proximal surfaces of the capitate and hamate. Capsular interposition or temporary fixation of the distal carpal row to the radius with K-wires will not be performed. However, radial styloidectomy may be performed according to the surgeon’s discretion.

### Four-corner arthrodesis

The scaphoid will be outright removed. The articular surfaces between the proximal row and distal carpal bones will be decorticated, and two to three cannulated compression screws will be used for fixation. In case of ulnar translation or dorsal intercalated segmental instability (DISI), the lunate will be re-aligned. The approach will be pragmatic to secure stable fixation in each case. Antegrade fixation of the luno-capitate space is not preferred to preserve the articular surface of the lunate. If adequate compression between these bones is not achievable using retrograde approach, an antegrade approach will be used. A cancellous bone autograft will be harvested from the removed scaphoid, the distal radius available through the same incision, or both.

### Rehabilitation

All participants will undergo an identical standardized postoperative rehabilitation for the first 12 postoperative weeks. The short arm splint applied in the operative theater and stitches will be removed 3 weeks postoperatively. Thereafter, the participants will perform active ROM exercises 3–5 times a day. A short arm removeable splint will be used until 6 weeks after the procedure. From 6 weeks onwards, the participants will perform active and passive ROM exercises and will be allowed to use the hand as tolerated. A physio- and/or occupational therapist specialized in treating hand surgery outpatients will instruct the participants at 3 and 6 weeks after the operation. After 12 weeks, any other rehabilitation input beyond the protocol will be at the discretion of the RIs.

Patient rehabilitation will be followed by RIs at an outpatient clinic. Both the patient and RI will be blinded to the allocation until 12 months post-surgery. Outcome measures collected during follow-up are presented in the “Outcomes {12}” section.

Cone beam computed tomography (CBCT) will be performed alongside the clinical follow-up at 6 and 12 weeks and 6 and 12 months after study procedure. To maintain blinding, the CBCT will be screened by the surgeon who will give permission to continue according to the rehabilitation program if no adverse events occur that would compromise the rehabilitation program. Neither RIs nor patients will have access to CBCT imagining before the primary endpoint of the study has been reached at 12 months. Long-term follow-up will include standard PA and lateral X-rays at 5 and 10 years after the surgery.

### Criteria for discontinuing or modifying allocated interventions {11b}

Crossover will not be possible due to the nature of the study procedures. Both interventions will be pragmatic to fit the needs of each participant. For instance, the surgeons will be allowed to perform radial styloidectomy during PRC if deemed necessary (radial styloid impingement). However, temporary fixation of the distal carpal row to the radius with K-wires will not be performed as this would necessitate wire removal and compromise the allocation. The diameter and length of the cannulated screws, insertion site, and screw direction will be left to the surgeons’ discretion. Nonetheless, a stable fixation is mandatory to allow early ROM exercises and further ensure the concealment during follow-up. If stable fixation is not reached, the patient will be excluded and the surgery and the rehabilitation will be performed according to the judgment of the treating surgeon.

If any concerns about patient safety arise in clinical examination or radiological evaluation during follow-up, the concealment will be unveiled, and the patient treated according to the standard of care.

### Strategies to improve adherence to interventions {11c}

Not applicable. Once performed, the trial interventions cannot be undone and crossover will not be possible.

### Relevant concomitant care permitted or prohibited during the trial {11d}

Not applicable. All trial patients will be treated according to standard of care.

### Provisions for post-trial care {30}

All trial patients will be treated according to standard of care. There is no anticipated harm that would differ from normal standard of care and no compensation for trial participation.

### Outcomes {12}

The primary endpoint of the study is PRWE at 1 year after the procedure. The secondary outcomes are Quick-Disabilities of Arm, Shoulder, and Hand (Q-DASH), pain on visual analog scale (VAS, 0 = no pain, 10 = worst imaginable pain), EQ-5D-5L, grip strength, wrist active ROM, global improvement, AEs, and radiologic evaluation (progress of arthritic changes, time to fusion in FCA participants) at the 1-year follow-up. Also, long term follow-ups (5 and 10 years) will be conducted.

The baseline data include patient age, gender, hand dominance, affected limb, duration of symptoms, smoking, education, type of work (desk-based to heavy manual), previous injuries, and surgery of the affected limb. Radiological baseline assessment includes wrist OA classification (SLAC/SNAC 0-IV), location of scaphoid non-union (distal, waist, proximal pole), ulnar variance, scapho-lunate- and scapho-capitate angle. Baseline clinical outcomes that will be assessed include ROM, and grip and key-pinch strength measurements.

The outcome variables and participant timeline are presented in Table 4.

**Table 4:**
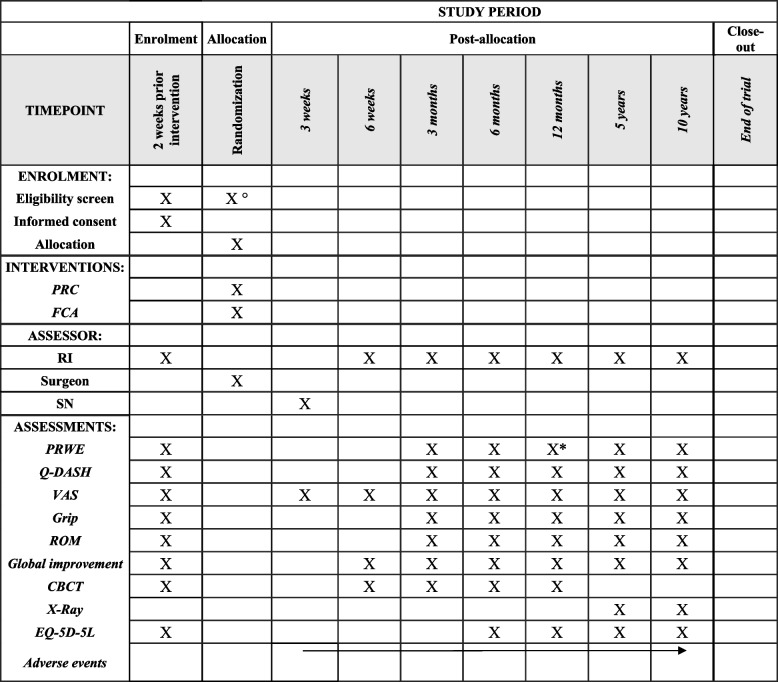
Schedule of enrolment, interventions, assessments, and data collection

*Primary endpoint

°Arthroscopy

*CBCT* Cone-beam computed tomography, *FCA* four-corner arthrodesis, *PRC* proximal row carpectomy, *PRWE* Patient-Rated Wrist Evaluation, *Q-DASH* Quick Disabilities of the Arm, Shoulder, and Hand, *RI* recruitment investigator, *ROM* range of motion, *SN* study nurse

### Primary outcome

#### Patient-Rated Wrist Evaluation

The Patient-Rated Wrist Evaluation (PRWE) is a patient-rated outcome measure for wrist and hand pathologies that is easy to administer and score in clinical practice. The PRWE is a 15-item questionnaire designed to measure pain and disability in activities of daily living [22]. The Finnish version has been translated, culturally adapted, and validated [23]. The score ranges from 0 to 100; a higher score indicates worse pain and function.

### Secondary outcomes

#### Quick Disabilities of the Arm, Shoulder, And Hand

The Quick-Disabilities of the Arm, Shoulder, and Hand (Q-DASH) is an upper extremity specific PROM with 11-items [24]. It is a widely used PROM for self-reported disability in various pathologies that affect the upper limb. Similar to the PRWE, the Finnish version has been translated, culturally adapted, and validated [25].

### Global improvement

Global improvement is a patient-centered standpoint of globally perceived benefit of the intervention. Global improvement is evaluated using five-step Likert scale from (− 2) “Much worse” to (+ 2) “Much better.”

### Pain

The visual analog scale (VAS) consists of a straight line with endpoints that define extreme limits to experiencing pain, from “no pain at all” and “worst possible pain” [26]. The subject is asked to mark their pain level on the line between the two endpoints. The distance (mm, 0 to 100) between “no pain at all” and the mark defines the subject’s pain. The VAS is a validated and reliable tool in pain assessment and is easy to use [27].

### Grip strength and wrist range of motion

Grip strength will be determined with a dynamometer (JAMAR hand dynamometer Model J00105, Lafayette, IN 47903, USA). Wrist active ROM will be measured using a manual goniometry following the American Society of Hand Therapists guidelines and Finnish Hand Therapy Societys guidelines [28, 29].

### Adverse events

Tendon, nerve, or arterial injury, chronic regional pain syndrome, infection, hematoma, deep vein thrombosis, non-union, implant-related complications, or any other conditions that can be attributed to the intervention will be regarded as an adverse event (event needing intervention or not resolving). Any complication leading to hospitalization, re-operation, or death will be considered as a major adverse event.

### Radiological evaluation

CBCT will be performed alongside the clinical follow-up at 6 and 12 weeks and 6 and 12 months. Moreover, standard PA and lateral view X-rays will be evaluated 5 and 10 years after study procedure. Time to union, non-union rates, implant-related complications, and development of postoperative OA will be recorded.

### EQ-5D-5L and cost-utility analysis

EQ-5D-5L is a standardized measure of health status developed by the EuroQol Group to provide a simple, generic measure of health for clinical and economic appraisal. Applicable to a wide range of health conditions and treatments, it provides a simple descriptive profile and a single index value for health status that can be used in the clinical and economic evaluation of health care. EQ-5D is designed for self-completion by respondents. It is cognitively undemanding and takes only a few minutes to complete [30]. In addition to health state evaluation, EQ-5D-5L will be used for the cost per quality-adjusted life year (QALY) gained evaluation after the procedure.

The costs will include treatment costs and possible costs of complications and sick leave. The costs of treatments and complications will be analyzed by collecting the actual use of healthcare services and multiplying the use of service by unit costs. Sick leaves and their societal costs will be collected from Social Insurance Institution of Finland (KELA).

### Participant timeline

Follow-up will take place at 3 and 6 weeks, at 3, 6, and 12 months, and at 5 and 10 years post-treatment. The trial schedule of enrollment, interventions, assessments, and data collection are presented in Table 4.

### Sample size {14}

The primary outcome measure is PRWE and the primary hypothesis of our trial is that PRC is non-inferior to FCA in the treatment of SLAC/SNAC OA I-II measured with PRWE total score. The non-inferiority margin is set at 11.5 points using the PRWE minimally clinically important difference [31]. To exclude the non-inferiority margin, the trial will require 37 patients in each group to observe MCID (non-inferiority margin 11.5, SD 14) in PRWE scores between the trial groups with a power of 90% and using a one-sided type I error rate of 2.5%. We will recruit 84 patients to account for 10% loss during follow-up.

### Recruitment {15}

We will assess the eligibility of all patients with SLAC/SNAC pattern wrist OA referred to the study hospitals. These participants will be referred to a RI and screened according to the inclusion and exclusion criteria. We estimate that the recruitment will be completed at the end of 2028.

## Assignment of interventions: allocation

### Sequence generation {16a}

A randomization sequence will be generated using an internet-based program (sealedenvelope.com). Patients will be allocated to one of the two treatment groups in a 1:1 ratio using permuted block randomization with variable block size.

### Concealment mechanism {16b}

The randomization will be performed by the research nurse by opening a sequentially numbered sealed opaque envelope after the surgeon has performed the wrist arthroscopy and confirmed the eligibility of the patient. As the envelopes will be kept in a secure, lockable cabinet that is only accessible by the study nurse located in the Helsinki University Hospital, an operating room nurse at each center will telephone the study nurse to receive and pass the knowledge of the allocation to the surgeon.

### Implementation {16c}

The RI, who is not involved in performing procedures, will enroll patients according to the inclusion and exclusion criteria. The interventions will be performed according to the randomization after the study surgeon has confirmed the eligibility by wrist arthroscopy. Randomization will be performed with the sealed opaque envelope method described above. The randomization sequence will be generated by an independent hand surgeon (Turkka Anttila, M.D.) familiar with clinical trial conduct and random sequence generation. Dr Anttila is not involved in the execution of the trial.

## Assignment of interventions: blinding

### Who will be blinded {17a}

The trial is patient- and outcome assessor (RI) -blinded. The patient will be blinded to the study intervention throughout the surgery by use of noise-canceling headphones. Moreover, patients will not have visual contact to the operation field or the surgeon performing the intervention. Operating room staff will not discuss the surgical method or disclose it to other hospital staff. No records revealing allocation will be accessible to the study patients.

The RI involved in patient enrolment and performing follow-up examinations will not participate in surgery and will not be able to access patient records containing knowledge of the treatment allocation. Follow-up CBCTs will be reviewed by the surgeon responsible for the intervention and concealed from the patient and RIs. If no (adverse) events compromising the standardized postoperative rehabilitation program are present in radiological evaluation during follow-up, the surgeon will give permission to continue the rehabilitation according to the study protocol.

The primary endpoint is PRWE score at 12 months post-intervention. This will also be the timepoint for unblinding.

### Procedure for unblinding if needed {17b}

In any case of a clinical situation that necessitates the knowledge of the trial group of the participant, patients can be unblinded (e.g., adverse event necessitating reoperation). However, whenever possible, the steering committee will discuss the clinical scenario and decide whether unblinding is necessary before it is performed. Each unblinding that occurs before assessment of primary endpoint will be reported.

## Data collection and management

### Plans for assessment and collection of outcomes {18a}

The RIs will collect all the baseline and follow-up data used for outcome reporting. All RIs are consultant level hand surgeons and will receive training in clinical measurements to improve inter-rater validity. A training log will be used to document all training completed by trial staff members. Moreover, each study center will receive an “investigator file” that contains all trial-related documentation and information to improve data collection.

Perioperative measures will be collected by the surgical team and include arthroscopic findings, type and number of cannulated screws, operation time (arthroscopy and total operative time), and time in the operating theater.

Postoperative outcome measures are discussed in detail in the “Outcomes {12}” section. The RIs will complete all clinical evaluations during follow-up and oversee the completeness of all questionnaire forms with a visual check of the responses. In case of incomplete questionnaires, RIs or SNs will inquire for missing data when possible.

### Plans to promote participant retention and complete follow-up {18b}

Participants may request unblinding at any point of the trial. However, to minimize unblinding before primary endpoint, the following will be addressed prior to trial enrolment to ensure that potential participants:

- Are willing to receive either of the interventions
- Are willing to remain with their allocation for 12 months

Unblinding prior to the 12 months does not affect the follow-up protocol or analyses.

### Data management {19}

A database including patients’ identification information and consent forms will include a unique identification code for each participant. A separate database where the participants are coded with the identification code will include the baseline and outcome data.

All RIs and SNs will be trained for trial electronic database use. When the questionnaire forms are received, the RI or SN will inspect the responses and inquire missing data when possible. The SN performing data entry will be blinded to group allocation. The forms will be stored into a password-protected electronic database on a hospital-provided server by means of double data entry to minimize typing errors. Patient records will be reviewed when collecting missing data or interpreting inconsistent or implausible data.

### Confidentiality {27}

All personal information about potential and enrolled participants are protected according to EU General Data Protection Regulation (GDPR). Case report forms (CRF) and all collected data from each trial center will be de-identified before creating a complete data set used for statistical analysis. No individual patient can be identified from the publication of trial results.

The full participant data will be stored for 12 months from the final conclusion of the study (after the 10-year follow-up visit). RIs are responsible of maintaining and ultimately destroying participant and processed data according to GDPR. Public access to the final trial dataset will be available on request from the principal investigator for research purposes based on steering committee assessment.

### Plans for collection, laboratory evaluation, and storage of biological specimens for genetic or molecular analysis in this trial/future use {33}

This trial does not involve collecting biological specimens for storage.

## Statistical methods

### Statistical methods for primary and secondary outcomes {20a}

Statistical analysis will be performed with both per-protocol and intention-to-treat (ITT) methods, the latter being the primary analysis. Descriptive statistics will be presented as mean (standard deviation) or median (interquartile range) for continuous variables and count (percent) for categorical variables.

For the continuous outcomes, we will compare the groups using a mixed model for repeated measurements (MMRM) entering group, group*time interaction as fixed factors, study site as a random factor, and baseline value as a covariate to the model. Marginal effects with 95% confidence intervals from the model will be reported as treatment effects. For the global improvement, we will use ordinal logistic model.

Statistical significance is set at 0.05. As comparisons of secondary outcomes are considered hypothesis generating, we will not adjust for multiple comparisons.

A statistical software program will be used for analyzing entered data. Blinded data interpretation will be used to reduce interpretation bias; therefore, the biostatistician will be unaware of the group assignments when performing the analyses.

The criterion for statistical significance will be set at *p* = 0.025 (one-sided) or *p* = 0.05 (two-sided). All *p*-values will be reported to three decimal places, with those less than 0.001 reported as *p* < 0.001.

### Interim analyses {21b}

For ethical and safety reasons, an interim analysis will be performed after enrolment of 42 (50%) patients. The purpose of this analysis is to ensure that the rates of adverse events are within acceptable limits (within the normal rate of complications related to PRC/FCA). The interim analysis will be carried out blinded to the group allocation unless a clear deviation in the incidence of AEs is found, in which case the allocation will be unveiled, and the study discontinued.

### Methods for additional analyses (e.g., subgroup analyses) {20b}

A cost-utility analysis (CUA) will be performed to compare financial aspects of FCA and PRC. The cost per quality-adjusted life year (QALY) method is used to compare the cost-utility ratio of FCA and PRC. The quality of health will be followed with EQ-5D-5L index and the change in this index at 1 year after surgery will be multiplied by the number of years spent in that health state to determine the number of QALYs gained or lost. To estimate QALYs gained or lost during patients’ lifetime, we will multiply the change in EQ-5D-5L index with the expected life years remaining. We will use the data from Official Statistics of Finland [32] to determine the life expectancy for each patient. We will use a constant discount rate of 5% when calculating the total QALYs gained over lifetime to account for a projected diminishing gain over time [33]. To calculate cost of QALY, we will divide the total expenses of FCA and PRC with the change of EQ-5D-5L. This will be adjusted to give the cost per QALY over the course of lifetime.

### Methods in analysis to handle protocol non-adherence and any statistical methods to handle missing data {20c}

We will document the number and proportion of patients eligible for and compliant with each follow-up. If the number of patients withdrawing from either arm of the trial is greater than the anticipated 10% at 12 months, an analysis of the demographic and prognostic characteristics will be performed between the individuals who withdraw and those who remain in the trial. Moreover, data may not be available due to voluntary withdrawal of patients, lack of completion of individual data items, or general loss to follow-up. For reliable ITT analyses, we will collect 1-year outcomes from all participants despite possible protocol compliance fluctuation. Where possible, the reasons for missing data will be ascertained and reported. However, the main analysis will be performed using the available (not imputed) data.

### Plans to give access to the full protocol, participant-level data, and statistical code {31c}

The datasets generated and analyzed during this trial will be available from the PI on reasonable request.

## Oversight and monitoring

### Composition of the coordinating center and trial steering committee {5d}

The Helsinki University Hospital will act as the study sponsor and coordinating center. The responsibility of the coordinating center is to provide necessary facilities for trial conduction.

A steering committee will be established prior to trial initiation. This committee will supervise trial execution and ensure that the trial is conducted according to the Medical Research Council’s (MRC) Guidelines for Good Clinical Practice.

### Composition of the data monitoring committee, its role and reporting structure {21a}

The Helsinki university central hospital (HUCH) institute is responsible for (1) clinical onsite monitoring according to EN14155 and maintaining (2) a written “investigator file” and (3) a monitoring manual to ensure patient’s rights, patient’s security, and reliability of trial results. The trial sites will be visited onsite by a clinical research associate before study initiation. During the trial, sites will be monitored at regular intervals depending on the rate of recruiting and data quality using onsite visits and video meetings. A monitoring log is used to document all visits completed by the research associate from HUCH institute.

Moreover, a data safety and monitoring committee (DSMC) will be established. The DSMC consists of a clinician familiar with SLAC/SNAC treatment, a clinician familiar with clinical trials, and a statistician. The DSMC will evaluate the safety of the trial based on major adverse event reporting.

### Adverse event reporting and harms {22}

AEs will be reported. Moreover, all major AEs will be documented and reported to the DSMC within 5 working days. All AEs occurring during trial participation will be treated according to standard of care.

### Frequency and plans for auditing trial conduct {23}

To ensure correct execution of the study, audits may be conducted if deemed necessary. However, routine audits are not planned.

### Plans for communicating important protocol amendments to relevant parties (e.g., trial participants, ethical committees) {25}

Any protocol amendments will be discussed by the study steering committee, reported to the Ethics Committee, and registered to ClinicalTrials.gov.

### Dissemination plans {31a}

The findings of this trial will be disseminated through peer-reviewed publications and conference presentations.

## Discussion

SLAC/SNAC of the wrist is a disabling progressive degenerative condition that is often treated surgically to reduce pain and improve function. Despite the growing number of scientific reports that address the matter, it is still unclear if FCA or PRC provides the most reliable outcome with fewer complications and less overall expenses. The present trial will compare these procedures in a high-quality setting to assist with clinical decision making when treating patients with this type of wrist OA.

PRC and FCA are common procedures for SLAC/SNAC wrist OA. Furthermore, SL-ligament injury and scaphoid non-union may present with irreducible carpal malalignment without significant degenerative changes. This type of condition is resistant to any attempt of reconstruction and will lead to an unfavorable outcome. Arguably, the pathomechanics of pain behind SL dissociation, scaphoid non-union, SLAC, and SNAC I-II wrist are different. Despite these nuances, a wrist surgeon will most likely treat these conditions similarly, namely with FCA or PRC. This trial will recruit patients presenting the different stages of SLAC/SNAC degenerative pattern provided they are not satisfied with non-surgical treatment regardless of the stage of the condition.

FCA was first described by Watson and Ballet in 1984 [2]. Since its introduction, numerous modifications have been described. Most commonly, the number of “corners” fused and implant(s) used will vary between techniques. Kirchner wires are inexpensive, easy to apply, and commonly used even though a rigid fixation cannot be achieved and pin removal is obligatory. Thus, many surgeons prefer more stable internal fixation with an implant(s) that does not necessitate removal (i.e., cannulated compression screws or a locking plate) [34–37].

Although a growing number of scientific papers have been published comparing these different techniques for FCA, no RCTs have been conducted. Based on non-randomized comparisons, the outcomes seem comparable [10, 15, 38, 39] and long-term outcomes of FCAs are reasonable [12, 40–42]. In this trial, we chose cannulated compression screws for rigid internal fixation to allow early ROM exercises and to ensure concealment of treatment allocation (no implant removal). All four “corners” will be decorticated and fused to achieve generalizable results. Cancellous bone graft is used to enhance ossifying potential [43, 44]. However, the placement, number, diameter, and length of the screws will be left to the surgeons’ discretion without compromising stability of the fixation.

PRC was described in 1944 by Stamm [45] and has regained popularity among surgeons treating wrist OA. The rationale for this procedure is similar to FCA, namely to preserve as much motion as possible while treating pain by relocating contact and motion of the wrist to a healthy articular cartilage. PRC is a rather easy procedure to perform and avoids the risk of bony non-union, implant loosening, breakage, and removal. The long-term outcomes of PRC have previously been shown to be rather good when compared to FCA long-term outcomes [16, 44, 46]. The possible disadvantages are mainly due shortened bony frame, which is hypothesized to cause impaired grip strength and impingement between the trapezium and radial styloid. Moreover, unlike FCA, PRC may not be performed on patients with advanced OA present at the head of the capitate, such in SLAC/SNAC III [19]. In this trial, patients with marked OA of the capitate head will be excluded. As with FCA, a pragmatic approach will be used for PRC, allowing the surgeon to perform radial styloidectomy when deemed necessary.

The pragmatic execution of the procedures will allow the surgeon to treat the patient according to the unique clinical presentation of the condition. However, this might also be interpreted as a limitation, as variation in the technique may potentially affect outcomes. However, we are not aware of any evidence that the modifications would affect the results and the pragmatic setting improves generalization. Another limitation is that the staff in the operation room cannot be blinded to the allocation. This is unlikely to cause performance bias as the surgeon and other operating room staff are excluded from postoperative treatment.

Two previous randomized trials comparing FCA and PRC exist. A study by Bisneto et al. [6] reported similar prospective outcomes after FCA vs PRC of 21 patients. There were several methodological issues with this study, namely risk of selection bias and imprecision of results. Moreover, the authors did not present a description of surgical technique or the number of patients treated with FCA or PRC. Aita et al. [7] presented results of a randomized trial comparing PRC (*n* = 13) vs FCA with a dorsal plate (*n* = 14). This study did not reveal statistically significant differences between study groups regarding postoperative pain, ROM, grip strength, or PROMs. The major drawback of this study was the small sample size, which makes it impossible to draw firm conclusions.

Besides these two prospective studies, numerous case series and retrospective studies have been conducted to find evidence to support the use of either FCA or PRC. Kamir et al. [17] included eight of these trials for their meta-analysis, which concluded that PRC is statistically superior to FCA when evaluating ROM, pain, and grip strength.

Despite the increasing number of scientific papers, there is still a paucity of evidence to guide clinical decision making due to the lack of high-quality RCTs. A rigorous RCT is the reference standard surgical trial design that attempts to limit sources of bias. Thus, we present an RCT design comparing FCA vs PRC in the treatment of SLAC/SNAC I-II OA by means of objective outcomes, PROMs, and complications in short- and long-term follow-up. The strength of this trial lies in its methodology and will provide high-quality data to aid decision making in the clinically common scenario of wrist OA.

### Trial status

The recruitment phase of the trial has started. The first participant was randomly assigned on 15 October 2020. Recruitment is expected to be completed by December 2024. This protocol is version 5.0, dated 26 November 2020. Trial completion is expected by December 2034.

### Supplementary Information

**Additional file 1.** 

## Data Availability

Public access to the final trial dataset will be available on request from the Principal Investigator for research purposes based on steering committee assessment.

## Abbreviations

AE: Adverse event
ASA: American Society of Anesthesiologists
ASHT: American Society of Hand Therapists
CBCT: Cone-beam computed tomography
CRF: Case report form
CUA: Cost-utility analysis
DISI: Dorsal intercalated segment instability
DSMC: Data safety and monitoring committee
EPL: Extensor pollicis longus
FCA: Four-corner arthrodesis
GDPR: General Data Protection Regulation
HUCH: Helsinki University Central Hospital
MCID: Minimal clinically important difference
MICE: Multiple Imputation by Chained Equations
MRC: Medical Research Council
OA: Osteoarthrosis
PA: Posteroanterior
PIN: Posterior interosseus nerve
PRC: Proximal row carpectomy
PRWE: Patient-Rated Wrist Evaluation
QALY: Quality-adjusted life year
Q-DASH: Quick Disabilities of the Arm, Shoulder, and Hand
RI: Recruitment investigator
ROM: Range of motion
SL: Scapholunar
SN: Study nurse
SLAC: Scapholunate advanced collapse
SNAC: Scaphoid non-union advanced collapse
VAS: Visual analog scale
