# Supplementary figures and images for: Proximal ROw carpectOmy versus four-corner Fusion (PROOF-trial) for osteoarthritis of the wrist: study protocol for multi-institutional double-blinded randomized controlled trial
Source: Trials. 2023 Aug 7;24:499. doi: 10.1186/s13063-023-07544-1 (PMC10405450; doi:10.1186/s13063-023-07544-1)

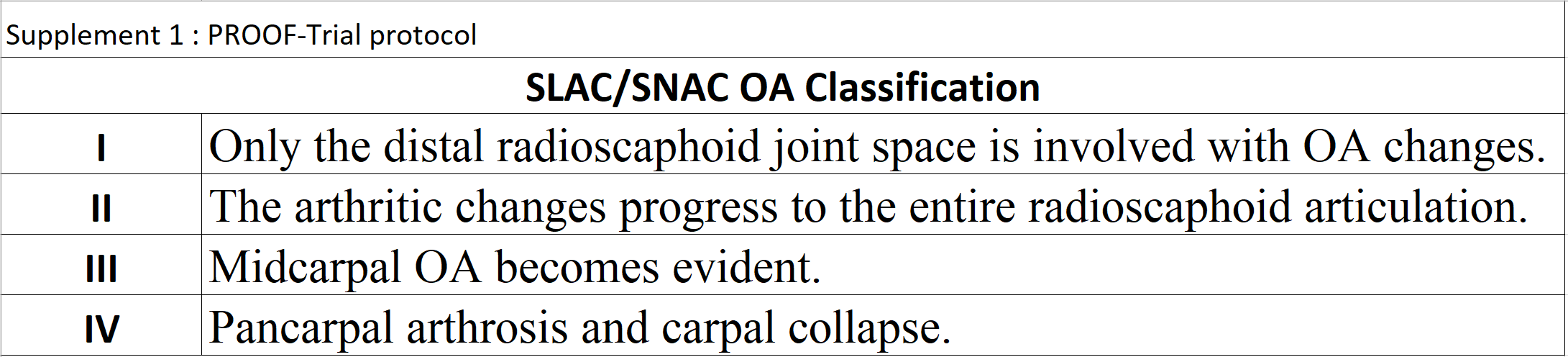

Supplement: Supplementary file 1 — Additional file 1. [file 13063_2023_7544_MOESM1_ESM.docx]
